# Methionine metabolism and endocrine function of the pituitary gland in patients with suprasellar germinoma

**DOI:** 10.1371/journal.pone.0288528

**Published:** 2023-07-13

**Authors:** Hwanhee Lee, Ji Won Lee, Hee Won Cho, Yearn Seong Choe, Kyung-Han Lee, Joon Young Choi, Ki Woong Sung, Seung Hwan Moon

**Affiliations:** 1 Department of Nuclear Medicine, Samsung Medical Center, Sungkyunkwan University School of Medicine, Seoul, Republic of Korea; 2 Department of Pediatrics, Samsung Medical Center, Sungkyunkwan University School of Medicine, Seoul, Republic of Korea; PGIMER: Post Graduate Institute of Medical Education and Research, INDIA

## Abstract

**Purpose:**

The aim of this study was to investigate the association between methionine (MET) metabolism and endocrine function of the pituitary gland in patients with suprasellar region tumor.

**Materials and methods:**

Twenty patients with intracranial germinoma were included in this study. Initial staging and all surveillance MET PET/CT scans and comparable serum levels of follicle stimulating hormone (FSH), luteinizing hormone (LH), and thyroid stimulating hormone (TSH) were analyzed. The patients were divided into two groups according to tumor location, with tumors in the suprasellar region (condition) or not (control). MET uptake of the pituitary gland (i.e., SUVR [standardized uptake value ratio]) and levels of FSH, LH, TSH were compared in the condition and control groups and in the before and after treatment phases of each group.

**Results:**

The SUVR in the control group was like that found in normal pituitary glands in previous studies, whereas the SUVR of the untreated condition group was high and that of treated condition group was low with significance compared to the control group. Serum levels of pituitary hormones in before and after treatment condition groups were significantly lower than those in the control group. The FSH and LH levels of curatively treated patients in the control group were positively correlated with SUVR with respective ß values of 3.71 and 0.98 (*p* < .001). The TSH level of the treated condition group was negatively correlated with SUVR (ß = -1.02, *p* < .001).

**Conclusion:**

This study is the first known investigation to examine the association between MET metabolism and endocrine function of the pituitary gland, and it confirmed that MET metabolism reflects endocrine function. A future study validating the result of correlation analysis is warranted.

## Introduction

Intracranial germinoma is an uncommon histologic type of brain tumor that occurs worldwide. However, the incidence of intracranial germinoma in Asian countries, including Korea, is higher than that in the West, especially the proportion of germinoma among pediatric brain tumors [1]. The diagnosis of intracranial germinoma is established by noninvasive diagnostic procedures such as neuroimaging, clinical manifestations, or laboratory findings including tumor marker, and biopsy is not required for a final diagnosis. Brain MRI is an essential diagnostic neuroimaging modality and C-11 methionine (MET) PET/CT is useful in tumor contouring, treatment planning, and response evaluation [2, 3]. The most common locations for intracranial germinoma are the pineal gland and the suprasellar region. The symptoms and laboratory findings typically depend on germinoma location; for tumors in the suprasellar region, common symptoms and laboratory findings are related to hypopituitarism because of the proximity of tumors to the pituitary gland [4].

Previous studies reported the findings of MET PET/CT in intracranial germinoma and endocrine disorders in patients with suprasellar tumors [3–5]. However, to the best of our knowledge, no previous studies have investigated the association between endocrine disorders and MET metabolism in patients with suprasellar germinoma. The aim of our study was to investigate the relationship between endocrine function and metabolic character on MET PET/CT in patients with intracranial germinoma, particularly suprasellar region tumor.

## Materials and methods

### Study cohort

Samsung Medical Center review board approved this retrospective study, and the requirement for informed consent was waived (2020-11-060-002). All procedures performed in our study were in accordance with the ethical standards of Samsung Medical Center research committee and with the 1964 Helsinki declaration and its later amendments or comparable ethical standards. We reviewed medical records from 66 consecutive patients who were diagnosed with intracranial germinoma and underwent brain C-11 MET PET/CT between 2014 and 2020. Of the 66 patients, 45 were excluded because they did not receive initial staging or surveillance MET PET/CT examination. With the hypothesis that MET metabolism of the pituitary gland and endocrine function are related, the included patients were divided into two groups according to the location of their tumor in the brain, i.e., whether the tumor accessed the pituitary gland (condition group) or not (control group). Nine patients with germinoma stemming from the pituitary gland (n = 1), suprasellar (n = 6), or infundibular region (n = 2) were categorized as the condition group. For the control group, 11 patients who had tumors in the pineal gland (n = 8) or basal ganglia (n = 3) were included. One patient had tumors in the pineal gland as well the pituitary gland and was excluded from the study.

**Fig 1:**
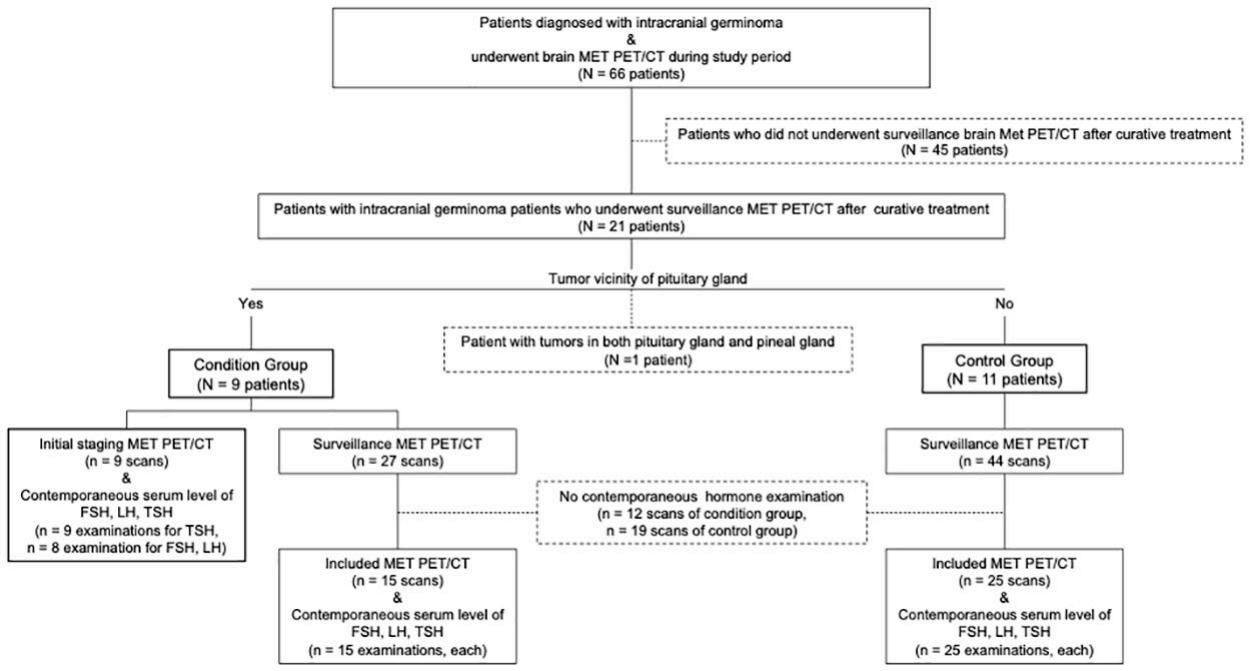
Inclusion and exclusion criteria flowchart of the cohort. * Dotted box: excluded subjects. FSH = follicle stimulating hormone, LH = luteinizing hormone, TSH = thyroid stimulating hormone.

The clinical examination results including brain MET PET/CT and serum levels of hormones produced by the pituitary gland were reviewed. Initial staging and surveillance MET PET/CT images of the 20 patients were included. The second and subsequent surveillance scans as well as the first routine follow-up PET scans were included (n = 71 scans). To investigate the relationship between MET uptake and endocrine function of the pituitary gland, the serum levels of hormones produced by the pituitary gland were studied within one month before or after each MET PET/CT imaging session. Comparable hormones analyzed were follicle stimulating hormone (FSH), luteinizing hormone (LH), and thyroid stimulating hormone (TSH). MET PET/CT images without contemporaneous serum levels of FSH, LH, or TSH were excluded (n = 31 scans). Fig 1 shows the flow of inclusion and exclusion criteria of the study cohort.

### Image acquisition and laboratory examinations

A dose of 6.0 MBq/kg C-11 labeled MET was intravenously injected in patients following at least four hours of fasting. Twenty minutes after injection, patients’ entire brains were scanned using a Discovery STE PET/CT scanner (GE Healthcare, Chicago, IL, US). CT scanning was performed with a continuous spiral technique and was followed by PET scanning for seven minutes per frame and reconstructed with an iterative three-dimensional reconstruction method. All laboratory tests were performed at our institution. Serum FSH and LH were measured using an immune-radiometric assay (DIAsource ImmunoAssays, Ottignies-Louvain-la-Neuve, Belgium), and radioactivity was detected by a fully automatic gamma counter (SR-300, STATEC, Luxembourg, Germany). TSH was measured using an immuno-radiometric assay (Beckman coulter, Pasadena, CA, US), and radioactivity was detected by a fully automatic gamma counter (Gamma Pro, Kaien, Seoul, Korea).

### Image analysis

The CT and PET scans were accurately co-registered using commercial software (Advantage Workstation VolumeShare 7/ AW 4.7, GE Healthcare, Chicago, IL, USA). The MET uptake of the region of interest was evaluated as maximal standardized uptake value (SUVmax). To evaluate MET uptake of the pituitary gland, a circular region of interest was drawn manually with reference to a non-contrast CT image of PET/CT. The SUVmax was automatically calculated by software as the product of pituitary gland activity concentration and administration dose of MET, divided by body weight. Four reference regions were chosen based on iso-metabolic activity with bilateral temporal and occipital cortices on the same plane with the pituitary gland on axial view. In our study, we defined MET uptake of the pituitary gland as a ratio of that of the pituitary gland to the mean of those of the four reference regions, i.e., SUVR.

### Statistical analysis

The Wilcoxon rank sum test and Wilcoxon signed rank test were conducted to compare the difference of SUVR and serum levels of FSH, LH, and TSH. Linear mixed effects models were used, and the correlation coefficient for repeated measures was estimated to assess the associations of SUVR with FSH, LH, and TSH [6]. To model the covariance structure, we considered simple, compound symmetric, unstructured, and autoregressive structures and selected the unstructured structure based on the smallest Akaike information criterion. Overall correlation coefficients between two variables can be considered for between-subject variability and within-subject variability. We also conducted Z tests to test the significance of the difference in coefficients between the control and condition groups and between pre- and post-treatment [7]. P values were two-sided, and the significance level was set at *p* < .05. All statistical analyses were performed using SAS version 9.4 (SAS Institute Inc., Cary, NC, USA) and R version 3.6.3.

## Results

### Patients

A total of 20 patients (male: female = 15: 5) with intracranial germinoma whose median age at diagnosis was 17 (10 to 31 years) was included. Of the nine suprasellar region germinoma patients, five were female and four were male, whereas all non-suprasellar germinoma patients were male. A summary of clinical characteristics of included patients is shown in Table 1.

**Table 1:** Patient characteristics.

Characteristics	Number of patients
Total	20
Male	15 (75%)
Female	5 (25%)
Median age at diagnosis (range)	16.5 (10–31)
Intracranial germinoma location	
Suprasellar region	6 (30%)
Pituitary region	3 (15%)
Pineal gland	8 (40%)
Basal ganglia	3 (15%)
Treatment	
Chemo-radiotherapy (Platinum + CSI 10.8 ~ 18 Gy + local boost 10.8 ~ 30.6 Gy)	19
Chemo-radiotherapy + craniotomy and tumor removal	1

### Methionine metabolism of the pituitary gland

The median MET uptake (SUVR) of the pituitary gland in the control group (n = 36 scans) was 1.85 (IQR, 0.31). The SUVR of the untreated control group (1.98 [IQR, 0.32], n = 11 scans) was higher than that of the post-treated control group (1.79 [IQR, 1.79], n = 25 scans) without significance (*p* = .28). The median MET uptake of the pituitary gland on initial MET PET/CT in the condition group (n = 9 scans) was 2.79 (IQR, 1.21), higher than that of the control group with significance (*p* < .001). The median MET uptake of the pituitary gland of on surveillance MET PET/CT in the condition group after curative treatment (n = 15 scans) was 1.10 (IQR, 0.19), significantly lower than that of the control group (*p* < .001) (Table 2).

**Table 2:** Median values of SUVR, FSH, LH, and TSH.

Variable	Total	N (cases)	Control	N (cases)	Condition	N (cases)	P-value
SUVR (median (IQR))							
All	1.81 (0.79)	60	1.85 (0.31)	36	1.42 (1.59)	24	0.196
Before treatment	2.05 (0.74)	20	1.98 (0.32)	11	2.79 (1.21)	9	0.112
After treatment	1.49 (0.64)	40	1.79 (0.16)	25	1.10 (0.19)	15	< 0.001
P-value	0.003		0.28		0.001		
FSH (median (IQR))							
All	2.10 (5.10)	57	3.70 (4.55)	34	0.30 (4.30)	23	0.01
Before treatment	1.50 (2.00)	17	1.70 (0.80)	9	0.30 (1.15)	8	0.06
After treatment	5.30 (8.10)	40	6.00 (4.65)	25	0.40 (5.50)	15	0.102
P-value	0.003		0.02		0.12		
LH (median (IQR))							
All	2.10 (1.80)	57	2.35 (1.10)	34	1.00 (0.60)	23	< 0.001
Before treatment	1.70 (1.60)	17	2.10 (1.00)	9	0.95 (0.88)	8	0.09
After treatment	2.25 (2.35)	40	3.20 (1.60)	25	1.00 (0.60)	15	0.001
P-value	0.30		0.16		0.93		
TSH (median (IQR))							
All	1.00 (1.99)	57	1.79 (0.93)	34	0.04 (0.32)	24	< 0.001
Before treatment	0.47 (1.84)	17	1.44 (1.64)	9	0.26 (1.20)	9	0.32
After treatment	1.31 (2.05)	40	2.05 (0.63)	25	0.03 (0.02)	15	< 0.001
P-value	0.58		0.07		0.20		

FSH = follicle stimulating hormone, LH = luteinizing hormone, TSH = thyroid stimulating hormone

### Serum levels of pituitary gland hormones; FSH, LH, and TSH

The median serum levels of FSH, LH and TSH of patients in the control group (n = 34 cases for each hormone) were 3.70 (IQR, 4.55), 2.35 (IQR, 1.10), and 1.79 (IQR, 0.93), respectively. In comparison between pre- and post-treatment in the control group, levels of LH and TSH were not significantly different: 2.10 (IQR, 1.00) vs. 3.20 (IQR, 1.60) (*p* = .16) for LH and 1.44 (IQR, 1.64) vs. 2.05 (IQR, 0.63) (*p* = .07) for TSH. However, for FSH, the median level of post-treated patients was significantly higher than that of untreated patients in the control group (6.00 [IQR, 4.65] vs. 1.70 [IQR, 1.70], *p* = .02). The median serum levels of FSH, LH, and TSH of patients in the condition group were lower than those in the control group regardless of treatment (*p* < .001). Respective median serum levels of FSH, LH, and TSH of untreated patients in the condition group (n = nine cases for TSH and eight cases for FSH and LH) were 0.30 (IQR, 1.15), 0.95 (IQR, 0.88) and 0.26 (IQR, 1.20), respectively. After curative treatment in the condition group (n = 15 cases for each hormone), the median levels of FSH, LH, and TSH were 0.40 (IQR, 5.50), 1.00 (IQR, 0.60) and 0.03 (IQR, 0.02), respectively (Table 2).

### Association between methionine metabolism and endocrine function of the pituitary gland

For correlation analysis between methionine uptake of the pituitary gland and the serum levels of pituitary gland hormones, a linear mixed model was applied to correct repeated measurements of a patient. The results showed that FSH and LH levels in the curatively treated control group were positively correlated with MET uptake of the pituitary gland, with ß values of 3.71 and 0.98, respectively (*p* < .001). The level of TSH in the curatively treated condition group was negatively correlated with MET uptake (ß = -1.02, *p* < .001). Table 3 summarizes the correlation between pituitary gland hormones and MET uptake of the pituitary gland (SUVR).

**Table 3:** Correlation between hormones and MET metabolism in the pituitary gland.

Variable	Total			Control			Condition			Z-statistics	
	ß (95% CI)	P-value	N (cases)	ß (95% CI)	P-value	N (cases)	ß (95% CI)	P-value	N (cases)	Z	P-value
**FSH**											
All	1.84 (1.04 to 2.65)	< 0.001	57	2.75 (1.93 to 3.58)	< 0.001	34	0.47 (-0.98 to 1.93)	0.53	23	2.71	0.01
Before treatment	-0.49 (-1.25 to 0.28)	0.23	17	0.23 (-0.48 to 0.94)	0.53	9	-1.33 (-2.69 to 0.04)	0.08	8	1.98	0.05
After treatment	2.89 (1.91 to 3.86)	< 0.001	40	3.71 (2.78 to 4.64)	< 0.001	25	1.51 (-0.57 to 3.60)	0.16	15	1.95	0.05
Z statistics	-5.35	< 0.001		-5.89	< 0.001		-2.29	0.02			
**LH**											
All	0.21 (0.01 to 0.57)	0.05	57	0.76 (0.51 to 1.02)	< 0.001	34	-0.40 (-0.89 to 0.07)	0.10	23	4.29	< 0.001
Before treatment	-0.46 (-0.97 to 0.05)	0.09	17	0.20 (-0.19 to 0.59)	0.33	9	-1.23 (-2.13 to -0.33)	0.02	8	2.84	0.01
After treatment	0.64 (0.38 to 0.90)	< 0.001	40	0.98 (0.71 to 1.24)	< 0.001	25	0.07 (-0.39 to 0.53)	0.77	15	3.49	< 0.001
Z statistics	-3.71	< 0.001		-3.24	0.001		-2.52	0.01			
**TSH**											
All	-0.61 (-0.91 to -0.31)	< 0.001	57	-0.22 (-0.50 to 0.05)	0.12	34	-1.18 (-1.67 to -0.69)	< 0.001	24	3.36	< 0.001
Before treatment	-0.10 (-1.70 to -0.27)	0.01	17	-0.60 (-1.29 to 0.10)	0.11	9	-1.44 (-2.76 to -0.12)	0.06	9	1.11	0.27
After treatment	-0.44 (-0.64 to -0.23)	< 0.001	40	-0.08 (-0.34 to 0.17)	0.53	25	-1.02 (-1.12 to -0.93)	< 0.001	15	6.90	< 0.001
Z statistics	-1.46	0.15		-1.41	0.16		-0.62	0.54			

FSH = follicle stimulating hormone, LH = luteinizing hormone, TSH = thyroid stimulating hormone

## Discussion

Intracranial germinoma patients in this study showed similar epidemiologic characteristics of previous Korean studies, which revealed that germinoma occurs primarily in teenagers, with no sex predominance in the suprasellar region and with male predominance in the pineal region [1].

In our retrospective study, patients with non-suprasellar germinoma including that after craniospinal irradiation were defined as the control group. Although there was some difference in method, MET uptake of a normal pituitary gland in a previous study (1.75 ± 0.17, mean SUV of pituitary gland ratio to normal frontal cortex uptake, 5–15 minutes (min) after injection using dynamic MET PET) [8] was similar to that of curatively treated patients in the control group of our study (SUVR = 1.79 [IQR, 0.16]). There are few previous studies using MET PET in intracranial germinoma. An earlier study evaluated the clinical efficacy of MET PET on germinoma in the central nervous system; there were two patients with suprasellar germinoma whose maximal SUV of tumor ratio to white matter were 5.7 (female, age 13 years) and 1.2 (male, age 29 years) [3], respectively, similar to the SUVR of untreated patients in the condition group of our study (range: 1.66 to 4.84).

The standard interpretation of a MET PET image in the region of interest relies on comparison with methionine uptake of a lesion with a normal value for that region, and uptake of the corresponding region in the contralateral hemisphere usually is measured as a normal value [9, 10]. In a region in the midline of the brain, such as the pituitary gland, it is difficult to identify a representative normal value. Previous studies used uptake of a normal frontal cortex and normal white or gray matter as the normal reference value and calculated tumor uptake ratio to uptake of normal reference [3, 8]. To approach an optimized representative reference value, we calculated a mean uptake of lesions in bilateral temporal and occipital white matter on the same plane as the pituitary gland on an axial image.

To the best of our knowledge, although it is a retrospective study with a small cohort size, this study is the first to investigate the association between MET metabolism and the endocrine function of the pituitary gland. The study demonstrated that MET uptake of the pituitary gland in untreated suprasellar germinoma, non-suprasellar germinoma, and curatively treated suprasellar germinoma was significantly different, with median SUVR values of 2.79 (IQR, 1.21), 1.85 (IQR, 0.31), and 1.11 (IQR, 0.19), respectively (*p* < .001). The serum levels of FSH, LH, and TSH of patients before and after treatment for suprasellar germinoma was significantly lower than those of patients with non-suprasellar germinoma; median levels were 0.30 (IQR, 1.15) and 0.40 (IQR, 5.50) vs. 3.70 (IQR, 4.55) for FSH (*p* < .001); 0.95 (IQR, 0.88) and 1.00 (IQR, 0.60) vs. 2.35 (IQR, 1.10) for LH (*p* < .001); and 0.26 (IQR, 1.20) and 0.03 (IQR, 0.02) vs. 1.79 (IQR, 0.93) for TSH (*p* < .001). The greater MET uptake of the pituitary gland in untreated condition group was cause by the presence of suprasellar tumor. In correlation analysis between MET metabolism and endocrine function of the pituitary gland, there was a positive correlation between FSH and SUVR (ß = 3.71, *p* < .001) and between LH and SUVR (ß = 0.98, *p* < .001) in curatively treated patients in the control group.

Extensively investigated but still a challenging problem in the interpretation of brain MET PET images is the differentiation between tumor recurrence and radiation necrosis after radiotherapy for brain tumor [2]. The uptake of MET as demonstrated on MET PET/CT reflects increased protein synthesis of a region of interest [11]. Our study initially started with a naïve expectation that if there is a certain trend between MET uptake of the pituitary gland and the level of pituitary hormone in a certain condition of patients with brain tumor in the vicinity of the pituitary gland, the trend might help in differentiating between tumor recurrence and radiation necrosis after radiotherapy. For example, an abnormally high MET uptake of the pituitary gland after curative treatment with a normal level of pituitary gland hormone favors pseudo progression, whereas abnormal hypermetabolism of MET with an abnormal (low) level of pituitary gland hormone favors true progression. However, in our study, even after curative treatment of suprasellar region germinoma, the level of pituitary gland hormone did not recover to the levels before disease. Some studies reported endocrine disorders in patients with curatively irradiated pituitary gland tumors, and a review article reported that growth hormone deficiencies can develop after a dose as low as 10 Gy, and that multiple hormone dysfunctions are common after 60 Gy irradiation [12]. In this study, patients received radiotherapy with a dose range of 21.6 Gy to 48.6 Gy. Another possible cause of the low level of MET uptake and hormones is prolonged compression by the tumor that may be small but remain. Therefore, the unrecovered low levels of MET uptake and FSH, LH, and TSH in patients with curatively treated suprasellar germinoma in our study were probably attributed to focal irradiation or prolonged compression of the pituitary gland [13].

Our study had several limitations. First, it was retrospective in design and included a small number of cases, which could result in relevant bias. Second, investigated hormones secreted by the pituitary gland were limited to FSH, LH, and TSH obtained from a one-off assessment rather than a dynamic test. Also, the serum levels of hormones including FSH and LH were not analyzed separately in female and male patients even though reference levels of FSH and LH are different according to sex and age. With these weaknesses, it is difficult to generalize or emphasize the results of correlation analysis between MET metabolism and hormone levels in the study. A future prospective study examining the correlation between MET uptake and endocrine function of the pituitary gland is warranted.

## Supporting information

S1 Data List of included patients and their clinical characteristics. (DOCX)
