# Supplementary material for: Methionine metabolism and endocrine function of the pituitary gland in patients with suprasellar germinoma
Source: PLoS One. 2023 Jul 13;18(7):e0288528. doi: 10.1371/journal.pone.0288528 (PMC10343025; doi:10.1371/journal.pone.0288528)
Supplement: S1 Data — (DOCX) [file pone.0288528.s001.docx]

Supplement Data

| Patient No. | Sex | Location of germinoma | Treatment | |  | PET No. | Age* | SUVR | Hormones | | |
| --- | --- | --- | --- | --- | --- | --- | --- | --- | --- | --- | --- |
|  |  |  |  |  |  |  |  |  | FSH | LH | TSH |
|  |  |  |  |  |  |  |  |  |  |  |  |
|  |  |  |  |  |  |  |  |  |  |  |  |
| Condition Group | | | | | | | | | | | |
|  |  |  |  |  |  |  |  |  |  |  |  |
| 1 | M | Infundibulum | Chemotherapy (Platinum) +  CSI 18Gy + local boost 12.6 Gy | |  | Tumor | 11 | 3.9 | 0.3 | 1.2 | 0.01 |
|  |  |  |  |  |  | 1 | 11 | 1.2 | 5.1 | 1.3 | 0.03 |
| 2 | F | Suprasellar | Chemotherapy (Platinum) +  CSI 18Gy + local boost 12.6 Gy | |  | Tumor | 18 | 3.0 | 0.1 | 1.1 | 0.346 |
|  |  |  |  |  |  | 2 | 18 | 0.99 | 0.3 | 0.6 | 0.1 |
|  |  |  |  |  |  | 3 | 19 | 1.1 | 0.3 | 1.0 | 0.04 |
| 3 | M | Infundibulum | Chemotherapy (Platinum) +  CSI 18Gy + local boost 12.6 Gy | |  | Tumor | 15 | 1.9 | 0.3 | 0.8 | 2.523 |
|  |  |  |  |  |  | 4 | 16 | 1.2 | 0.1 | 1.8 | 0.01 |
|  |  |  |  |  |  | 5 | 16 | 1.2 | 0.1 | 0.4 | 0.01 |
| 4 | F | Suprasellar | Chemotherapy (Platinum) +  CSI 18Gy + local boost 12.6 Gy | |  | Tumor | 11 | 2.79 | - | - | 0.56 |
|  |  |  |  |  |  | 6 | 11 | 1.06 | 0.2 | 0.8 | 0.013 |
|  |  |  |  |  |  | 7 | 12 | 1.02 | 0.1 | 1.0 | 0.024 |
| 5 | F | Suprasellar | Chemotherapy (Platinum) +  CSI 18Gy + local boost 12.6 Gy | |  | Tumor | 10 | 1.12 | 0.3 | 0.6 | 0.017 |
|  |  |  |  |  |  | 8 | 11 | 4.84 | 0.3 | 0.5 | 0.41 |
|  |  |  |  |  |  | 9 | 11 | 1.26 | 0.2 | 1.1 | 0.019 |
|  |  |  |  |  |  | 10 | 12 | 0.92 | 0.2 | 0.8 | 0.022 |
| 6 | F | Suprasellar | Chemotherapy (Platinum) +  CSI 18Gy + local boost 12.6 Gy | |  | Tumor | 31 | 3.11 | 0.5 | 0.8 | 5.109 |
|  |  |  |  |  |  | 11 | 32 | 1.21 | 0.4 | 0.7 | 0.005 |
| 7 | M | Suprasellar | Chemotherapy (Platinum) +  CSI 18Gy + local boost 12.6 Gy | |  | Tumor | 19 | 3.55 | 0.1 | 0.6 | 0.177 |
|  |  |  |  |  |  | 12 | 19 | 1.11 | 1.8 | 0.5 | 0.013 |
|  |  |  |  |  |  | 13 | 20 | 1.01 | 5.7 | 0.6 | 0.056 |
| 8 | M | Pituitary gland | Chemotherapy (Platinum) +  CSI 10.8Gy + local boost 10.8 Gy | |  | Tumor | 12 | 1.66 | 4.1 | 3.0 | 0.94 |
|  |  |  |  |  |  | 14 | 13 | 1.62 | 8.8 | 2.8 | 1.23 |
| 9 | F | Suprasellar | Chemotherapy (Platinum) +  CSI 10.8Gy + local boost 10.8 Gy | |  | Tumor | 19 | 2.39 | 4.5 | 2.9 | 0.18 |
|  |  |  |  |  |  | 15 | 19 | 0.97 | 16.1 | 4.0 | 0.04 |
|  |  |  |  |  |  |  |  |  |  |  |  |
|  |  |  |  |  |  |  |  |  |  |  |  |
| Control Group | | | | | | | | | | | |
|  |  |  |  |  |  |  |  |  |  |  |  |
| 1 | M | Basal ganglia | Chemotherapy (Platinum) +  CSI 18Gy + local boost 12.6 Gy | |  | 1 | 16 | 1.84 | 3.5 | 2.4 | 2.1 |
|  |  |  |  |  |  | 2 | 17 | 1.97 | 1.9 | 2.4 | 1.5 |
|  |  |  |  |  |  | 3 | 18 | 1.37 | 0.8 | 2.0 | 1.4 |
|  |  |  |  |  |  | 4 | 19 | 1.75 | 1.1 | 2.6 | 1.8 |
|  |  |  |  |  |  | 5 | 20 | 1.72 | 0.8 | 2.3 | 1.8 |
| 2 | M | Pineal gland | Chemotherapy (Platinum) +  CSI 18Gy + local boost 30.6 Gy | |  | 6 | 16 | 2.65 | 6.3 | 2.7 | 1.6 |
|  |  |  |  |  |  | 7 | 17 | 1.87 | 5.5 | 4.4 | 1.6 |
| 3 | M | Pineal gland | Chemotherapy (Platinum) +  CSI 18Gy + local boost 30.6 Gy +  Craniotomy and tumor removal | |  | 8 | 17 | 1.99 | 6.5 | 3.6 | 0.6 |
|  |  |  |  |  |  | 9 | 18 | 1.78 | 4.1 | 3.8 | 1.7 |
| 4 | M | Pineal gland | Chemotherapy (Platinum) +  CSI 18Gy + local boost 12.6 Gy | |  | 10 | 25 | 1.69 | 5.8 | 3.0 | 1.4 |
|  |  |  |  |  |  | 11 | 25 | 1.32 | 6.0 | 3.2 | 1.0 |
| 5 | M | Pineal gland | Chemotherapy (Platinum) +  CSI 18Gy + local boost 12.6 Gy | |  | 12 | 21 | 2.08 | 8.8 | 3.9 | 2.4 |
| 6 | M | Pineal gland | Chemotherapy (Platinum) +  CSI 18Gy + local boost 12.6 Gy | |  | 13 | 14 | 1.64 | 7.8 | 2.0 | 2.7 |
|  |  |  |  |  |  | 14 | 14 | 2.13 | 9.5 | 2.6 | 1.3 |
|  |  |  |  |  |  | 15 | 15 | 1.89 | 9.2 | 2.2 | 2.9 |
| 7 | M | Pineal gland | Chemotherapy (Platinum) +  CSI 18Gy + local boost 12.6 Gy | |  | 16 | 20 | 1.63 | 4.4 | 1.9 | 1.7 |
|  |  |  |  |  |  | 17 | 21 | 2.02 | 4.3 | 3.0 | 1.7 |
|  |  |  |  |  |  | 18 | 21 | 1.35 | 1.7 | 2.1 | 1.4 |
| 8 | M | Basal ganglia | Chemotherapy (Platinum) +  CSI 18Gy + local boost 12.6 Gy | |  | 19 | 13 | 1.81 | 4.4 | 3.0 | 1.4 |
|  |  |  |  |  |  | 20 | 14 | 1.78 | 5.2 | 1.9 | 2.4 |
|  |  |  |  |  |  | 21 | 14 | 1.79 | 4.6 | 2.4 | 2.1 |
| 9 | M | Pineal gland | Chemotherapy (Platinum) +  CSI 18Gy + local boost 12.6 Gy | |  | 22 | 16 | 1.69 | 10.2 | 3.2 | 0.2 |
|  |  |  |  |  |  | 23 | 17 | 1.79 | 9.3 | 1.9 | 2.2 |
| 10 | M | Basal ganglia | Chemotherapy (Platinum) +  CSI 18Gy + local boost 12.6 Gy | |  | 24 | 19 | 2.09 | 9.7 | 4.3 | 2.3 |
| 11 | M | Pineal gland | Chemotherapy (Platinum) +  CSI 10.8Gy + local boost 10.8 Gy | |  | 25 | 23 | 1.93 | 6.9 | 3.2 | 2.3 |
|  | | | | | | | | | | | |

⁎ Age at the time of imaging

M = male; F = female; CSI = craniospinal irradiation; FSH = follicle stimulating hormone; LH = luteinizing hormone; TSH = thyroid stimulating hormone
